# Supplementary material for: Molybdenum isotopes unmask slab dehydration and melting beneath the Mariana arc
Source: Nat Commun. 2021 Oct 14;12:6015. doi: 10.1038/s41467-021-26322-8 (PMC8517010; doi:10.1038/s41467-021-26322-8)
Supplement: Supplementary file 1 — Supplementary Information [file 41467_2021_26322_MOESM1_ESM.pdf]

Supplementary Information

Molybdenum isotopes unmask slab dehydration and melting beneath the Mariana arc

Hong-Yan Li[1, 2, 3*], Rui-Peng Zhao[1, 4], Jie Li[1, 2], Yoshihiko Tamura[5], Christopher Spencer[6], Robert J. Stern[7], Jeffrey G. Ryan[8], Yi-Gang Xu[1, 2, 3]

[1] *State Key Laboratory of Isotope Geochemistry, Guangzhou Institute of Geochemistry, Chinese Academy of Sciences, Guangzhou 510640, China*

[2] *CAS Center for Excellence in Deep Earth Science, Guangzhou, 510640, China*

[3] *Southern Marine Science and Engineering Guangdong Laboratory (Guangzhou), Guangzhou, 511458, China*

[4] *University of Chinese Academy of Sciences, Beijing 100049, China*

[5] *Research Institute for Marine Geodynamics (IMG), Japan Agency for Marine-Earth Science and Technology (JAMSTEC), Yokosuka 237-0061, Japan*

[6] *Department of Geological Sciences and Geological Engineering, Queen's University, Kingston, ON K7L 3N6, Canada*

[7] *Department of Geoscience, University of Texas at Dallas, Richardson, TX 75080, United States*

[8] *School of Geosciences, University of South Florida, Tampa, FL 33620, United States*

1. Supplementary Methods

Trace element analyses of the serpentinites were performed at Guizhou Tongwei Analytical Technology Co., Ltd. on a Thermal X series 2 equipped with a Cetac ASX-510 AutoSampler. Approximately 60 mg of each sample powder was dissolved in a Teflon bomb with a doubly distilled concentrated HF-HNO_3 (4:1) mixture. The dissolution was maintained in an oven at 185 °C for 3 days. The solutions were then evaporated, and the sample residues were re-dissolved with double distilled concentrated HNO_3 and evaporated again to convert the fluorides from the initial HF dissolution to nitrates. Then, the samples were dissolved in a final 3ml 2M HNO_3 stock solution. Finally, sample solutions were diluted with 2% HNO_3 to achieve a 4000 × dilution factor. An internal spike consisting of 12ppb ^{6}Li, 6ppb ^{61}Ni, Rh, In, and Re, and 4.5ppb ^{235}U was added to the sample solutions to monitor instrumental drift. USGS standard W-2a was used as a reference standard and crossed checked with BIR-1 and BHVO-2. Instrument drift mass biases were corrected with internal spikes and external monitors. The ICP-MS procedure for trace element analysis follows the protocol of Eggins et al.[1] with modifications as described in Kamber et al.[2] and Li et al.[3].

2. Supplementary Table

Supplementary Table 1 Input parameters for the geochemical modeling and calculated slab components for the Mariana arc lavas

**Input parameters**	DM1 (Depleted DMM)	DM2 (Enriched DMM)	MORB	AMOC	Sediments	Average eclogite and blueschist
Mo (μg/g)	0.014 [a]	0.024 [a]	0.46 [c]	0.37 [e]	2.49 [h]	0.17 [k]
Ce (μg/g)	0.421 [a]	0.726 [a]	14.86 [c]	11.40 [f]	34.30[i]	16.05 [k]
Nd (μg/g)	0.483 [a]	0.703 [a]	12.03 [c]	11.30 [f]	25.20 [i]	13.16 [k]
Hf (μg/g)	0.127 [a]	0.186 [a]	2.79 [c]	3.07 [f]	1.44 [i]	2.82 [k]
$\delta^{98/95}$Mo (‰)	-0.21 [b]	-0.21[b]	-0.21 [b]	0.36 [e]	-0.29 [h]	-0.51 [k]
^{143}Nd/^{144}Nd	0.513080 [d]	0.513025 [d]		0.513118 [g]	0.512310 [i]	
^{176}Hf/^{177}Hf	0.283220 [d]	0.283180 [d]		0.283164 [g]	0.282897 [j]	

**Slab components**	Shallow Fluid 700 ℃	Slab Melt 4 GPa	Deep Lithosphere Fluid	Slab Melt 6 GPa
Mo (μg/g)	2.46 [l]	1.17 [o]	5.07 [p]	1.06 [q]
Ce (μg/g)	1.69 [l]	50.08 [o]	1.87 [p]	53.92 [q]
Nd (μg/g)	0.71 [l]	18.26 [o]	0.68 [p]	30.21 [q]
Hf (μg/g)	0.05 [l]	1.24 [o]	0.05 [p]	1.14 [q]
$\delta^{98/95}$Mo (‰)	0.25 [m]	-0.40 [o]	0.06 [p]	-0.07 [q]
^{143}Nd/^{144}Nd	0.512958 [n]	0.512958 [n]		0.512958 [n]
^{176}Hf/^{177}Hf	0.283151 [n]	0.283151 [n]		0.283151 [n]

[a] The Ce-Nd-Hf of the depleted mantle are from Workman and Hart[4]. Mo is estimated according Ce/Mo = 30. DM: depleted mantle; DMM:

depleted mid-ocean ridge basalt (MORB) mantle.

[b] Depleted mantle from Bezard et al.[5].

[c] Average MORB from Gale et al.[6].

[d] Estimated according the "ambient mantle" for the Izu-Bonin-Mariana arc from Woodhead et al.[7].

[e] ODP Site 801 altered mafic oceanic crust (AMOC) super composite from Freymuth et al.[8].

[f] ODP Site 801 AMOC super composite from Kelley et al.[9].

[g] ODP Site 801 AMOC super composite from Chauvel et al.[10].

[h] Average Mariana sediment (ODP Sites 800, 801, 802) from Freymuth et al.[8].

[i] Average ODP Site 1149 sediment from Plank et al.[11].

[j] Bulk composition of ODP Site 1149 sediment from Chauvel et al.[10].

[k] Average composition of eclogite and blueschist from Chen et al.[12]. Samples SEC43-1 and SEC43-1 are not included.

[l] Trace element contents calculated for shallow slab fluid at 700 °C (90% AMOC + 10% Sediments; F=2%), applying batch dehydration model. Partition coefficients for Ce-Nd-Hf are from Kessel et al.[13] at 700 °C, 4 GPa. The Mo content is calculated according the uniform Ba/Mo=230 for the Pagan Northeastern Flank samples and the calculated Ba content for the fluid.

[m] Shallow fluid Mo isotope composition from Villalobos-Orchard et al.[14].

[n] Bulk mixing of 90% AMOC + 10% Sediments, constrained according the Hf-Nd isotope and Hf/Nd covariations of the Pagan samples.

[o] Trace element contents calculated for model batch melting of the subducted slab (90% AMOC + 10% Sediments) at 900 °C with F=10%. Partition

coefficients for Ce-Nd-Hf are from Kessel et al.[13] at 900 °C, 4 GPa. Partition coefficients for Mo are from Adam and Green[15] and Chen et al.[12], considering a 2 wt.% rutile in the source and a ratio between clinopyroxene and garnet of 70:30. Mo isotopes calculated according Mo isotope equilibrium fractionation factor of $\Delta^{98/95}Mo_{melt\text{-}rutile}$ = 0.5‰ at 900°C between the melt and the residual rutile. The Mo isotope fractionation factor is calculated according the experimental result of $\Delta^{98/95}Mo_{melt\text{-}rutile}$ = 0.33 ± 0.06‰ at 1175°C between the melt and the residual rutile[12].

[p] Trace element contents calculated for fluid from the deep slab lithosphere at 700 °C, applying batch dehydration model and F=2%. The deep lithosphere fluid is assumed to equilibrate with an eclogite source that has experienced 2% fluid percolation and Mo loss at shallow depth. Partition coefficients for Ce-Nd-Hf are from Kessel et al.[13] at 700 °C, 4 GPa. Partition coefficient for Mo calculated after Bali et al.[16] at 700 °C, 2.61 GPa, oxygen fugacity of FMQ+ 4 and NaCl content of 5 wt.%, considering a 2 wt.% rutile in the source and a ratio between clinopyroxene and garnet of 70:30. Mo isotopes calculated according Mo isotope equilibrium fractionation factor of $\Delta^{98/95}Mo_{fluid\text{-}rutile}$ = 0.73‰ at 700°C between the fluid and the residual rutile. The Mo isotope fractionation factor is calculated according the experimental result of $\Delta^{98/95}Mo_{melt\text{-}rutile}$ = 0.33 ± 0.06‰ at 1175°C between the melt and the residual rutile[12].

[q] Trace element contents calculated for model batch melting of deep fluid fluxed slab (2% Deep fluid+98% slab) at 900 °C with F=10%. The slab is assumed to have experienced early melting at 4 GPa (900 °C; F=10%). Partition coefficients for Ce-Nd-Hf from Kessel et al.[13] at 900 °C, 6 GPa. Partition coefficient for Mo from Adam and Green[15] and Chen et al.[12], considering a 2 wt.% rutile in the source and a ratio between clinopyroxene and garnet of 70:30. Mo isotopes calculated according Mo isotope equilibrium fractionation factor of $\Delta^{98/95}Mo_{melt\text{-}rutile}$ = 0.5‰ at 900°C between the melt and the residual rutile. The Mo isotope fractionation factor is calculated according the experimental result of $\Delta^{98/95}Mo_{melt\text{-}rutile}$ = 0.33 ± 0.06‰ at 1175°C between the melt and the residual rutile[12].

3. Supplementary Figures

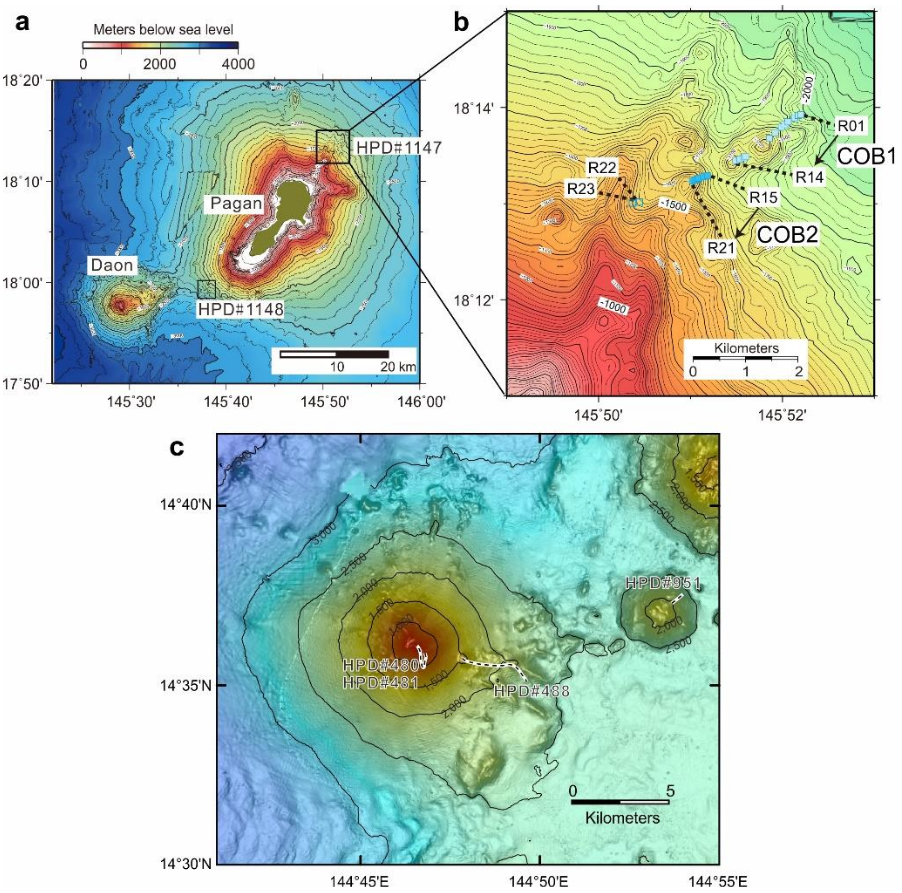

Supplementary Fig. 1 Volcanoes of this study from the Mariana Bathymetric Compilation with sampling locations. a Pagan and Daon volcanoes from the Mariana Bathymetric Compilation showing the locations of ROV Hyper-Dolphin dives during cruise NT10-12[17]: the Northeastern Flank of Pagan (HPD1147) and the Southern Flank of Pagan (HPD1148). **b** Bathymetry of Pagan's northeastern slopes showing HPD1147 dive tracks[17]. During this dive, two distinct types of primitive clinopyroxene-olivine basalt (COB1 and COB2) lavas were collected only 500m apart. **c** NW Rota-1 volcano showing the sampling tracks of ROV Hyper-Dolphin[18]: the Summit (HPD 480 and 481), Eastern Flank (HPD 488), and East Knoll (HPD 951). COB: clinopyroxene-olivine basalt; POB: plagioclase-olivine basalt.

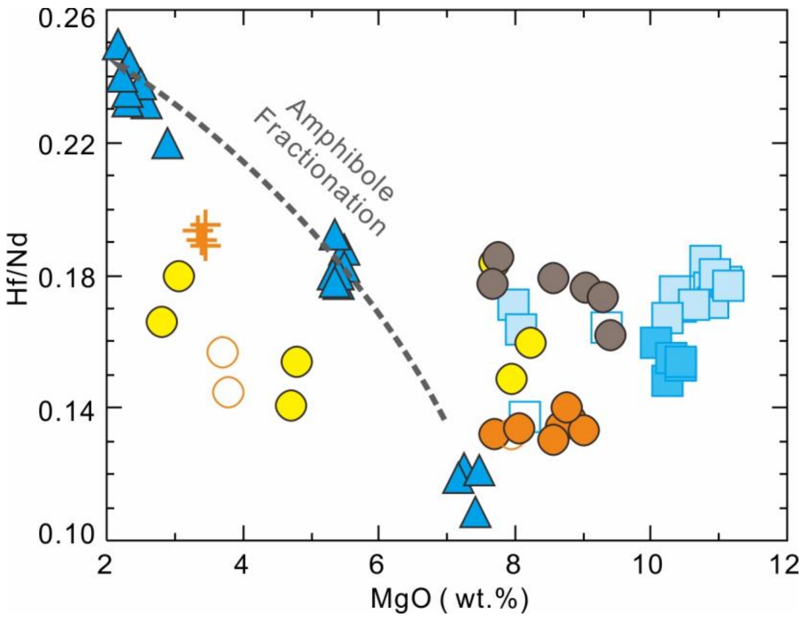

Supplementary Fig. 2 Hf/Nd versus MgO diagram for Pagan and NW Rota-1 samples.

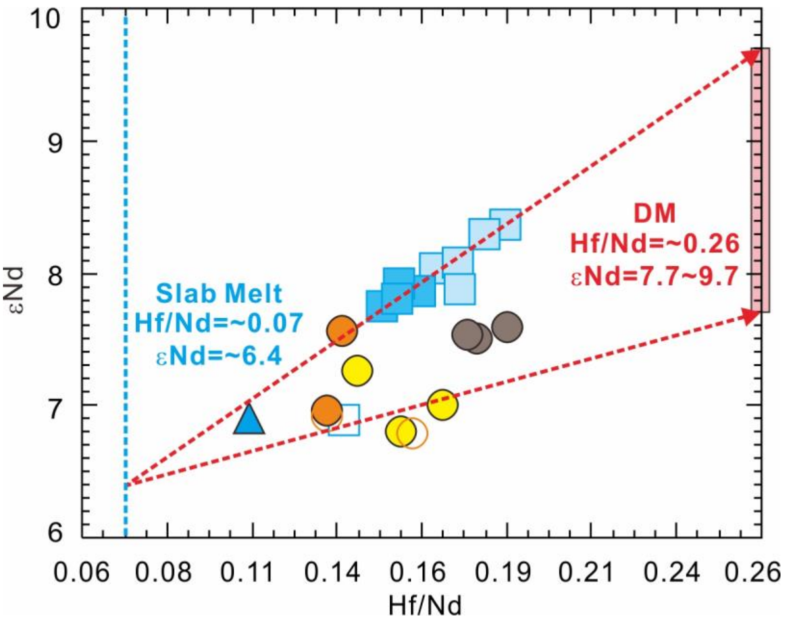

Supplementary Fig. 3 εNd versus Hf/Nd diagram used to estimate the εNd and Hf/Nd of the slab melt and ambient mantle. The slab melt is estimated to have εNd of ~ 6.4 and Hf/Nd of 0.07. Then the slab surface is constrained to be composed of altered mafic oceanic crust and sediments with a ratio of 9:1. The ambient mantle is estimated to have εNd between 7.7 and 9.7. DM: depleted mantle.

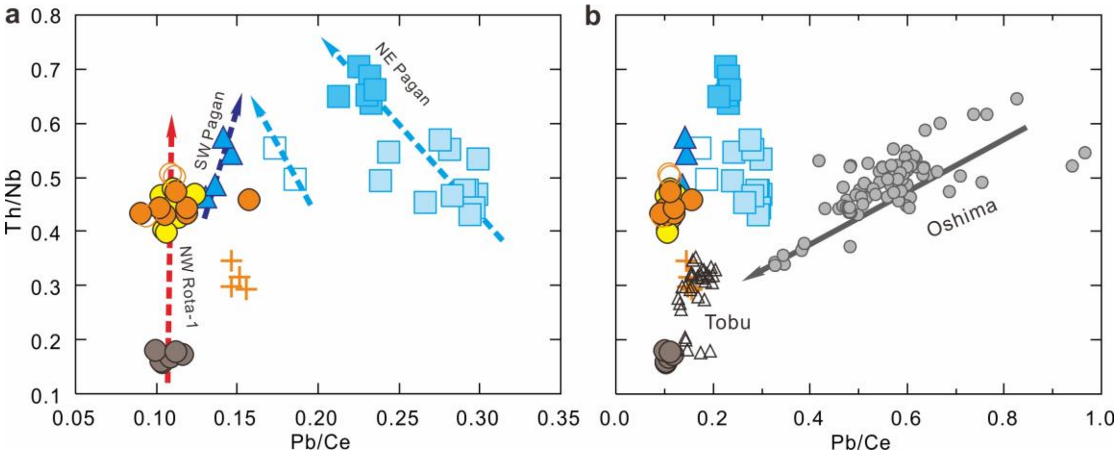

Supplementary Fig. 4 Th/Nb versus Pb/Ce diagrams for Pagan and NW Rota-1 samples unaffected by amphibole fractional crystallization. Izu-Oshima and Izu-Tobu samples[19] are also plotted for comparison in diagram **b**. The variation of the Izu-Oshima (volcanic front) samples is explained by addition of Izu-Tobu (backarc) magma to the Izu-Oshima plumbing system. This model is difficult to explain the geochemistry of Pagan samples. Note the high Pb/Ce and high Th/Nb characteristics indicate the slab fluid for the Oshima volcano may be supercritical. It is different with that for the Pagan volcano.

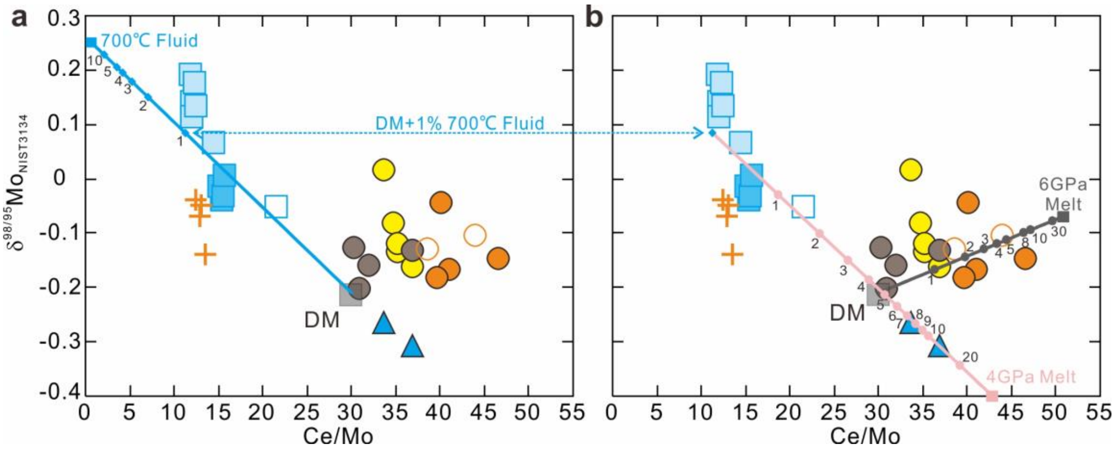

Supplementary Fig. 5 $\delta^{98/95}$Mo versus Ce/Mo diagrams for Pagan and NW Rota-1 samples unaffected by amphibole fractional crystallization, showing slab dehydration/melting process. The depleted mantle (DM), 700 °C slab fluid, 4 GPa slab melt and 6 GPa slab melt compositions are listed in the Supplementary Table 1. The numbers on the mixing curves between different compositions represent the mass percentage of the slab fluid/melt. Blue line in **a** is the mixing trend between DM and the 700℃ slab fluid. Pink line in **b** is the mixing trend between the partially serpentinized mantle (DM + 1% 700 °C fluid) and the 4 GPa slab melt. Gray line in **b** is the mixing trend between the DM and the 6 GPa slab melt.

Supplementary References

1. Eggins, S. M. et al. A simple method for the precise determination of ≥40 trace elements in geological samples by ICPMS using enriched isotope internal standardisation. *Chem. Geol.* **134**, 311–326 (1997).
2. Kamber, B. S., Greig, A., Schoenberg, R. & Collerson, K. D. A refined solution to Earth's hidden niobium: implications for evolution of continental crust and mode of core formation. *Precambrian Res.* **126**, 289–308 (2003).
3. Li, B. P. et al. ICP-MS trace element analysis of Song dynasty porcelains from Ding, Jiexiu and Guantai kilns, north China. *J. Archaeol. Sci.* **32**, 251–259 (2005).
4. Workman, R. K. & Hart, S. R. Major and trace element composition of the depleted MORB mantle (DMM). *Earth Planet. Sci. Lett.* **231**, 53–72 (2005).
5. Bezard, R., Fischer-Gödde, M., Hamelin, C., Brennecka, G. A. & Kleine, T. The effects of magmatic processes and crustal recycling on the molybdenum stable isotopic composition of Mid-Ocean Ridge Basalts. *Earth Planet. Sci. Lett.* **453**, 171–181 (2016).
6. Gale, A., Dalton, C. A., Langmuir, C. H., Su, Y. J. & Schilling, J.-G. The mean composition of ocean ridge basalts. *Geochem. Geophys. Geosyst.* **14**, 489–518 (2013).
7. Woodhead, J. D., Stern, R. J., Pearce, J. A., Hergt, J. & Vervoort, J. Hf-Nd isotope variation in Mariana Trough basalts: The importance of "ambient mantle" in the interpretation of subduction zone magmas. *Geology* **40**, 539–542 (2012).
8. Freymuth, H., Vils, F., Willbold, M., Taylor, R. N. & Elliot, T. Molybdenum mobility and isotopic fractionation during subduction at the Mariana arc. *Earth Planet. Sci. Lett.* **432**, 176–186 (2015).
9. Kelley, K. A., Plank, T., Ludden, J. & Staudigel, H. Composition of altered oceanic crust at ODP Sites 801 and 1149. *Geochem. Geophys. Geosyst.* **4**, 8910 (2003).
10. Chauvel, C., Marini, J.-C., Plank, T. & Ludden, J. N. Hf-Nd input flux in the Izu-Mariana subduction zone and recycling of subducted material in the mantle. *Geochem. Geophys. Geosyst.* **10**, Q01001 (2009).
11. Plank, T., Kelley, K. A., Murray, R. W. & Stern, L. Q. Chemical composition of sediments subducting at the Izu-Bonin trench. *Geochem. Geophys. Geosyst.* **8**, Q04I16 (2007)
12. Chen, S. et al. Molybdenum systematics of subducted crust record reactive fluid

flow from underlying slab serpentine dehydration. *Nat. Commun.* **10**, 4773 (2019).

13. Kessel, R., Schmidt, M. W., Ulmer, P. & Pettke, T. Trace element signature of subduction-zone fluids, melts and supercritical liquids at 120–180 km depth. *Nature* **437**, 724–727 (2005).
14. Villalobos-Orchard, J. et al. Molybdenum isotope ratios in Izu arc basalts: The control of subduction zone fluids on compositional variations in arc volcanic systems. *Geochim. Cosmochim. Acta* **288**, 68–82 (2020).
15. Adam, J. & Green, T. Trace element partitioning between mica- and amphibole-bearing garnet lherzolite and hydrous basanitic melt: 1. Experimental results and the investigation of controls on partitioning behaviour. *Contrib. Mineral. Petrol.* **152**, 1–17 (2006).
16. Bali, E., Keppler, H. & Audetat, A. The mobility of W and Mo in subduction zone fluids and the Mo–W–Th–U systematics of island arc magmas. *Earth Planet. Sci. Lett.* **351–352**, 195–207 (2012).
17. Tamura, Y. et al. Mission immiscible: distinct subduction components generate two primary magmas at Pagan volcano, Mariana arc. *J. Petrol.* **55**, 63–101 (2014).
18. Tamura, Y. et al. Two primary basalt magma types from Northwest Rota-1 volcano, Mariana arc and its mantle diapir or mantle wedge plume. *J. Petrol.* **52**, 1143–1183 (2011).
19. Ishizuka, O. et al. Progressive mixed-magma recharging of Izu-Oshima volcano, Japan: A guide to magma chamber volume. *Earth Planet. Sci. Lett.* **430**, 19–29 (2015).
