# Supplementary material for: Molybdenum isotopes unmask slab dehydration and melting beneath the Mariana arc
Source: Nat Commun. 2021 Oct 14;12:6015. doi: 10.1038/s41467-021-26322-8 (PMC8517010; doi:10.1038/s41467-021-26322-8)
Supplement: Supplementary file 2 — Description of Additional Supplementary Files [file 41467_2021_26322_MOESM2_ESM.pdf]

Description of Additional Supplementary Files

File name: Supplementary Data 1.

Description: Major elements, trace elements and Sr-Nd-Pb-Hf-Mo isotopes for the Pagan and NW Rota-1 volcanic rock samples and Asùt Tesoru serpentinite mud samples.
